# Association of rural living with COPD-related hospitalizations and deaths in US veterans

**DOI:** 10.1038/s41598-023-34865-7

**Published:** 2023-05-16

**Authors:** Spyridon Fortis, Yubo Gao, Arianne K. Baldomero, Mary Vaughan Sarrazin, Peter J. Kaboli

**Affiliations:** 1 Veterans Rural Health Resource Center-Iowa City, VA Office of Rural Health, and Center for Access and Delivery Research and Evaluation (CADRE) at the Iowa City VA Healthcare System, Iowa City, IA USA; 2 Department of Internal Medicine, Division of Pulmonary, Critical Care, and Occupational Medicine, University of Iowa Roy J. and Lucille A. Carver College of Medicine, Iowa City, IA USA; 3 Department of Internal Medicine, Division of General Internal Medicine, University of Iowa Roy J. and Lucille A. Carver College of Medicine, Iowa City, IA USA; 4 Minneapolis VA Health Care System US, Minneapolis, MN USA; 5 Pulmonary, Allergy, Critical Care, and Sleep Medicine, University of Minnesota, Minneapolis, MN USA

**Keywords:** Epidemiology, Respiratory tract diseases

## Abstract

It is unclear whether the high burden of COPD in rural areas is related to worse outcomes in patients with COPD or is because the prevalence of COPD is higher in rural areas. We assessed the association of rural living with acute exacerbations of COPD (AECOPDs)-related hospitalization and mortality. We retrospectively analyzed Veterans Affairs (VA) and Medicare data of a nationwide cohort of veterans with COPD aged ≥ 65 years with COPD diagnosis between 2011 and 2014 that had follow-up data until 2017. Patients were categorized based on residential location into urban, rural, and isolated rural. We used generalized linear and Cox proportional hazards models to assess the association of residential location with AECOPD-related hospitalizations and long-term mortality. Of 152,065 patients, 80,162 (52.7%) experienced at least one AECOPD-related hospitalization. After adjusting for demographics and comorbidities, rural living was associated with fewer hospitalizations (relative risk-RR = 0.90; 95% CI: 0.89–0.91; P < 0.001) but isolated rural living was not associated with hospitalizations. Only after accounting for travel time to the closest VA medical center, neighborhood disadvantage, and air quality, isolated rural living was associated with more AECOPD-related hospitalizations (RR = 1.07; 95% CI: 1.05–1.09; P < 0.001). Mortality did not vary between rural and urban living patients. Our findings suggest that other aspects than hospital care may be responsible for the excess of hospitalizations in isolated rural patients like poor access to appropriate outpatient care.

## Introduction

Chronic obstructive pulmonary disease (COPD) patients experience acute exacerbations of the disease (AECOPDs), defined as worsening of their respiratory symptoms that results in additional therapy^1^. Severe AECOPDs, defined as those requiring emergency room visit or hospitalization, are associated with increased mortality^2–7^ and are responsible for up to 70% of the direct health care costs of the disease^8,9^. Although, AECOPDs typically occur more frequently as the disease progresses, there is significant variation in exacerbation susceptibility among patients. One such group, the “frequent exacerbator phenotype” defined as patients with COPD that experience ≥ 2 exacerbations per year, are responsible for up to half of all hospitalizations^10,11^.

Another group at high risk for worse health outcomes is people living in rural areas. AECOPD-related hospitalizations rates are 21% higher among individuals living in rural areas relative to those living in urban areas in United States. The AECOPD-related death rates are also 70% higher in rural living individuals relative to urban living individuals^12^. The rural–urban disparity in COPD could result from the poor access to care in rural patients with COPD but could also be due to the higher prevalence of COPD in rural areas. The COPD prevalence in rural areas is 74% higher than the COPD prevalence in urban areas. In a US study that included only patients with COPD, rural living was a risk factor for AECOPDs, but was not a risk factor for AECOPD-related hospitalizations^13^. Patients with COPD may choose not go to the hospital when they reside far away from it. It is unclear whether the high burden of COPD in rural areas is related to worse outcomes in patients with COPD or is because the prevalence of COPD is higher in rural areas. The study goal was to compare AECOPD-related hospitalizations and mortality between urban and rural patients with COPD. We studied a cohort of veterans diagnosed with COPD who received care in Veterans Affairs (VA) and community hospitals using merged VA and Medicare data. We assessed the association of rural living with AECOPD-related hospitalizations, “the frequent exacerbator phenotype”, and mortality among patients with established COPD. To further investigate the association of rural living with the aforementioned outcomes, we created several multivariable models with different sets of co-variates including travel time to the nearest VA medical center, area deprivation index (ADI), a measure of neighborhood socioeconomic disadvantage^14,15^, and air pollution.

## Methods

This retrospective cohort study included VA patients aged 65 years or older with at least two encounters (inpatient and/or outpatient) with a principal diagnosis of COPD between October 1, 2011 and September 30, 2014, with available data for at least one year prior to the second COPD encounter. Cohort entry was defined as the time of the second encounter. Using VA administrative data and Medicare claims data, we retrieved all AECOPD-related hospitalizations at acute care VA or community hospitals during the study period with follow up to September 30, 2017. The Institutional Review Board and Research and Development Committee at the Iowa City VA Health Care System [IRB 201712713] has approved this study and waived informed consent. This is work is a part of a larger mixed-methods study described in a previous publication^16,17^. The methods carried out in this study may have overlap with our previous work^16^. All methods were carried out in accordance with relevant guidelines and regulations. The study followed the Strengthening the Reporting of Observational Studies in Epidemiology (STROBE) guidelines for observational studies^18^.

### Setting

We obtained data from the Veterans Informatics and Computing Infrastructure (VINCI), an integrated system that includes VA's electronic health records and administrative data. Admissions to VA acute care hospitals were identified via the Corporate Data Warehouse using the inpatient domain. These datasets contain patient demographics including residential address and ZIP code, diagnosis and procedure codes during admission, admission source, and admission and discharge dates. Data regarding non-VA AECOPD-related hospitalizations were obtained from the Centers for Medicare and Medicaid Services (CMS) administrative data and Non-VA Medical Care (Fee Basis) data. Data used for the study were covered under a data use agreement with CMS and are not available for distribution by the authors but available from CMS.

### Definitions

COPD diagnosis was defined based on the following International Classification of Diseases, Ninth and Tenth Revisions, Clinical Modification codes: (ICD-9-CM: 490.x 491.xx, 492.xx, and 496.xx or ICD-10-CM: J41, J43, J43.1, J43.2, J43.8, J43.9, J44.0, J44.1, J41.8, J42 or J44.9). Patient residential location was defined using census tracts based on Rural Urban Commuting Area (RUCA) codes^16^. RUCA codes reflect measures of urbanization, commuting, and population density^19–23^. RUCA codes were further categorized into: urban (1 and 1.1), rural (2, 2.1, 3, 4, 4.1, 5, 5.1, 6, 7, 7.1, 7.2, 8, 8.1, 8.2, and 9), and isolated rural (10, 10.2, 10.2, and 10.3) as defined by the VA Office of Rural Health^24^. According to that, urban area is a land with at least 30% of the population residing in a continuously built-up area with a population of ≥ 50,000 inhabitants. Isolated rural area is a sparsely populated land with < 10% of the working population commutes to a larger community with ≥ 2500 but < 50,000 inhabitants. Rural areas are those lands that are not defined as urban or isolated rural. Travel time to the nearest VA hospital was determined from VA Planning Systems and Support Group geo-coded enrollment files. Distance is calculated to the nearest VA hospital using actual longitude and latitude coordinates of patient residences with travel time estimated using geospatial technologies taking into account roads and average driving conditions^19^. Comorbidities were defined based on the corresponding ICD-9-CM and ICD-10-CM diagnosis codes within 1 year prior to the insertion of the cohort^16^. The ADI ranks neighborhoods by socioeconomic disadvantage, based on income, education, employment, and housing quality using data from the American Community Survey 5-year estimates^14,15^. The ADI defines neighborhood as census block group, which we aggregated to census tracts. Particulate matter 2.5 (PM_2.5_) was used as a surrogate of ambient air quality (pollution) and was calculated using Environmental Protection Agency (EPA) data for years 2013–2018^25^.

### Outcomes

The primary outcome, AECOPD-related hospitalization, is defined using the following criteria: 1) a principal diagnosis of COPD or 2) a principal diagnosis of acute respiratory failure (ICD-9-CM 518.81, 518.82, 518.84, or 799.1; ICD-10-CM: J96.00, J96.01, J96.02, J96.11, J96.12, J96.20, J96.21, J96.22, J96.90, J96.92 or R06.03) with a secondary AECOPD diagnosis (ICD-9-CM: 491.21, 491.22; ICD-10-CM: J44.1, J44.0) as previously described^16,19^. The secondary outcomes are the presence of “frequent exacerbator phenotype” defined as patients with COPD that have ≥ 2 AECOPD-related hospitalizations per year, and death defined using the date of death from the VA Vital Status File between COPD diagnosis and September 30, 2017.

### Statistical analysis

We categorized patients with COPD based on residential location into urban, rural, and isolated rural. Comparison of characteristics was made between groups using ANOVA for continuous variables and Chi-squared for categorical variables. We created generalized linear models with a Poisson distribution to assess the association of residential location with hospitalizations per year. We created three multivariable models: (1) Model 1 included age, sex, race, obstructive sleep apnea, diabetes mellitus, congestive heart failure, coronary artery disease, cancer, chronic kidney disease, and an offset variable, defined as the log of the length of follow-up time (time from COPD diagnosis to Death or the end of the study) to account for the varying follow-up periods, as the covariates in the model, (2) Model 2 included all the covariates of Model 1 plus travel time to the nearest VA hospital as a continuous variable, and (3) Model 3 included all the covariates of Model 2 plus ADI and PM_2.5_ as continuous variables. We also created generalized linear models with a logit link to assess the association of residential location with the “frequent exacerbator phenotype”. All multivariable models included the following co-variates: age, sex, race, obstructive sleep apnea, diabetes mellitus, congestive heart failure, coronary artery disease, cancer, and chronic kidney disease. We created three multivariable models as above (Model 1, Model 2 = Model 1 + Travel time, Model 3 = Model 2 + ADI + PM_2.5_). Cox proportional hazards regression analysis was employed to examine the association of patient residential location and mortality. Most patients in our cohort had zero or one AECOPD-related hospitalizations and were at low risk for death. If we included all patients with COPD, the outcome (death) would be rare and the analysis will no longer be clinically relevant. In addition, the increase of mortality associated with each additional AECOPD-related hospitalization is relatively stable after the second hospitalization^26^. For those reasons, we limited the mortality analysis to only those survived after the second AECOPD-related hospitalization. For this analysis, the cohort entry was defined as the date of live discharge from the hospital after the second AECOPD-related hospitalization. We created three multivariable models as in the “the frequent exacerbator phenotype” analysis. All statistical analysis were conducted using SAS Enterprise Guide, 2014 SAS Institute Inc.

## Results

We included a total of 152,065 patients aged 65 and older with at least two COPD encounters between October 1, 2011, and September 30, 2014. The median follow-up time was 982 days (Interquartile Interval = 453–1440 days). Of these 152,065 patients, 80,162 (52.7%) experienced at least one AECOPD-related hospitalizations between the entry cohort and September 2017(end of the study). Approximately, one third of AECOPD-related hospitalizations occurred outside the VA health care system. Supplement Table 1 shows the count of non-VA AECOPD-related hospitalizations and the count of total hospitalizations (AECOPD-related and non-AECOPD-related) in urban, rural, and isolated rural veterans. Approximately 17.6% of patients in the cohort experienced ≥ 1 AECOPD-related hospitalization per year and were responsible for 50.0% of total AECOPD-related hospitalizations; 9.7% of patients experienced ≥ 2 AECOPD-related hospitalizations per year (“frequent exacerbator phenotype”) and were responsible for 27.0% of all AECOPD-related hospitalizations. The number of patients by AECOPD-related hospitalization frequency are shown in Table 1.

**Table 1:** Distribution of COPD patients by AECOPD-related hospitalization frequency (n = 152,065).

	Hospitalizations per year							
	0	> 0 to < 1	≥ 1 to < 2	≥ 2 to < 3	≥ 3 to < 4	≥ 4 to < 5	≥ 5 to < 6	≥ 6
Patient count	71,903 (47.3%)	53,376 (35.1%)	12,112 (8.0%)	4527 (3.0%)	2268 (1.5%)	1415 (0.9%)	1025 (0.7%)	5439 (3.6%)
Total hospitalizations	0	71,553	32,981	15,004	7424	4445	2964	8820
Patient rurality								
Urban	41,078 (57.1%)	31,981 (59.9%)	7456 (61.6%)	2817 (62.2%)	1397 (61.6%)	869 (61.5%)	657 (64.1%)	3355 (61.7%)
Rural	26,087 (36.3%)	18,299 (34.3%)	4036 (33.3%)	1468 (32.4%)	746 (32.9%)	484 (34.2%)	316 (30.8%)	1768 (32.5%)
Isolated	4738 (6.6%)	3096 (5.8%)	620 (5.1%)	242 (5.4%)	125 (5.5%)	62 (4.4%)	52 (5.1%)	316 (5.8%)

AECOPD, acute exacerbation of COPD.

Characteristics of patients categorized by residential location are described in Table 2. Obstructive sleep apnea, diabetes mellitus, and coronary artery disease were more prevalent among rural and isolated rural patients relative to urban while congestive heart failure, cancer, and chronic kidney disease were more prevalent in urban individuals. The average ADI was higher and the average annual PM_2.5_ was lower in rural and isolated rural groups relative to urban group. The travel time to the nearest VA medical center was longer for rural and isolated rural patients relative to urban individuals.

**Table 2:** Characteristics of COPD patients stratified by residential location (n = 152,065).

	Urban (n = 89,610)	Rural (n = 53,204)	Isolated Rural (n = 9,251)	P value*
Age, y	75.41 ± 0.03	74.50 ± 0.03	74.91 ± 0.08	< 0.0001
Sex(female), n(%)	1650 (1.84%)	684 (1.29%)	122 (1.32%)	< 0.0001
Race, n (%)				< 0.0001
White	73,259 (81.75%)	49,934 (93.85%)	8892 (96.12%)	
Black	14,814 (16.53%)	2512 (4.72%)	168 (1.82%)	
Other	1537 (1.72%)	758 (1.42%)	191 (2.06%)	
Obstructive sleep Apnea, n (%)	13,883 (15.49%)	8470 (15.92%)	1560 (16.86%)	0.0008
Diabetes Mellitus, n (%)	34,911 (38.96%)	21,257 (39.95%)	3683 (39.81%)	0.0006
Congestive Heart Failure, n (%)	24,640 (27.50%)	14,205 (26.70%)	2481 (26.82%)	0.0034
Coronary Artery Disease, n (%)	37,920 (42.32%)	23,307 (43.81%)	3978 (43.00%)	< 0.0001
Cancer, n (%)	20,846 (23.26%)	11,395 (21.42%)	1926 (20.82%)	< 0.0001
Chronic Kidney Disease, n (%)	19,152 (21.37%)	10,190 (19.15%)	1766 (19.09%)	< 0.0001
Travel Time to VA, min	75.66 ± 0.45	128.12 ± 0.57	182.40 ± 1.92	< 0.0001
Area Deprivation Index	57.71 ± 0.09	67.20 ± 0.08	70.61 ± 0.18	< 0.0001
Annual PM2.5, 10 μg/m^3^	9.15 ± 0.01	8.74 ± 0.01	8.04 ± 0.02	< 0.0001
Hospitalization /year	1.83 ± 0.05	1.60 ± 0.06	1.77 ± 0.16	0.0116

Data are presented as number (%) or mean ± stander error.

*ANOVA for continuous variables and Chi-squared for categorical variables.

### AECOPD-related hospitalizations

**Figure 1:**
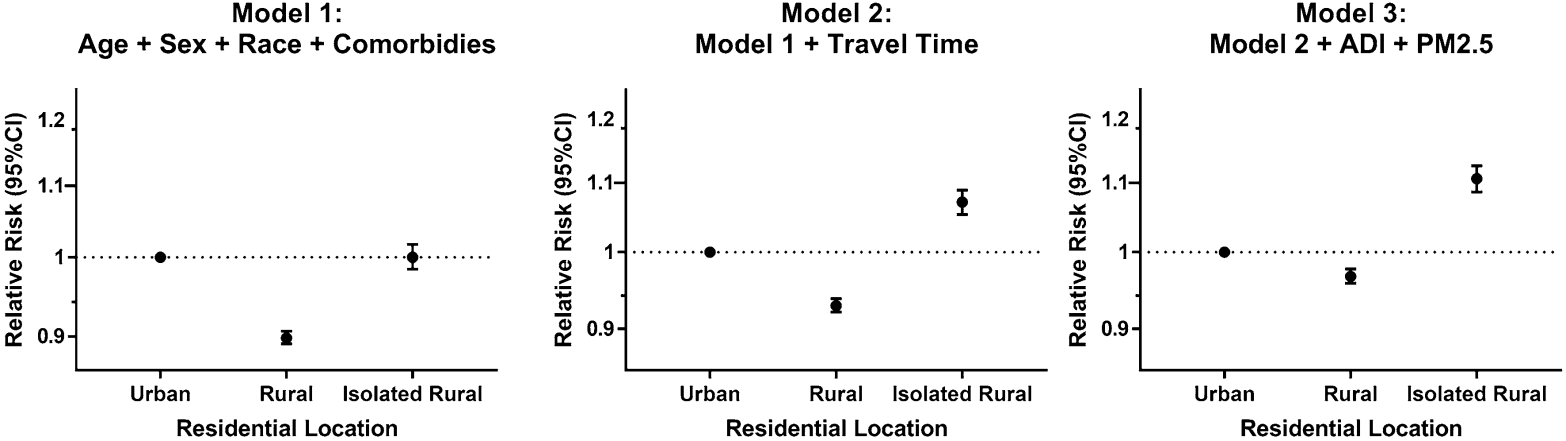
Association of residential location with AECOPD-related hospitalization frequency (hospitalizations/year) in COPD patients (n = 152,065). We created generalized linear models with a Poisson distribution to assess the association of residential location with AECOPD-related hospitalizations. All models included the following co-variates: age, sex, race, obstructive sleep apnea, diabetes mellitus, congestive heart failure, coronary artery disease, cancer, chronic kidney disease, and an offset variable defined as the log of the length of follow-up time (time from COPD diagnosis to Death or the end of the study) to account for the varying follow-up periods. AECOPD, acute exacerbation of COPD; RR, relative risk.

The unadjusted average AECOPD-related hospitalizations/year were 1.83 ± 0.05 [standard error (SE)] in urban, 1.60 ± 0.06 (SE) in rural, and 1.77 ± 0.16 (SE) in isolated rural group (Table 2). In univariate analysis, both isolated rural and rural living were associated with fewer AECOPD-related hospitalizations compared to urban. After adjusting for age, sex, race, and comorbidities, rural living was associated with fewer (relative risk (RR) = 0.90; 95% CI: 0.89–0.91; P < 0.001) but isolated rural living (RR = 1.00; 95% CI: 0.98–1.02; P = 0.96) was not associated with AECOPD-related hospitalizations (Fig. 1). After adding travel time to the nearest VA medical center in the model, rural living was associated with fewer AECOPD-related hospitalizations (RR = 0.93; 95% CI: 0.92–0.94; P < 0.001) but isolated rural living was associated with more AECOPD-related hospitalizations (RR = 1.07; 95% CI: 1.05–1.09; P < 0.001). This association did not change after adding ADI and PM_2.5_ in the model.

### Frequent-exacerbator phenotype

**Figure 2:**
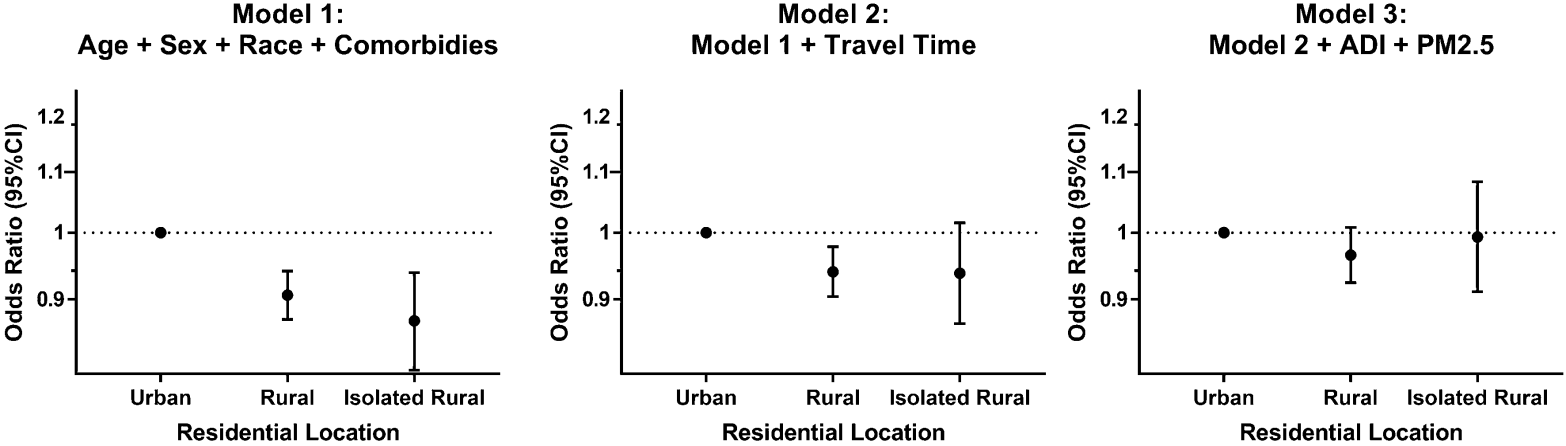
Association of residential location with “frequent exacerbator phenotype”, defined as patients that had ≥ 2 AECOPD-related hospitalizations/year (n = 152,065). We created generalized linear models with a logit link to assess the association of residential location with frequent exacerbator phenotype, defined as patients that had ≥ 2 AECOPD-related hospitalizations/year. All models included the following co-variates: obstructive sleep apnea, diabetes mellitus, congestive heart failure, coronary artery disease, cancer, and chronic kidney disease. AECOPD, acute exacerbation of COPD; OR, odds ratio.

In univariate analysis, both isolated rural and rural living (compared to urban) were inversely associated with the “frequent exacerbator phenotype”. In the multivariable analysis, after adjusting for age, sex, race, and comorbidities, both isolated rural living (odds ratio (OR) = 0.87; 95% CI: 0.81–0.94; P < 0.001) and rural living (OR = 0.91; 95% CI: 0.87–0.94; P < 0.001) was inversely associated with the “frequent exacerbator phenotype” relative to urban residence (Fig. 2). These associations became weaker after adding travel time to the model and disappeared after adding ADI and PM_2.5_.

### Mortality

**Figure 3:**
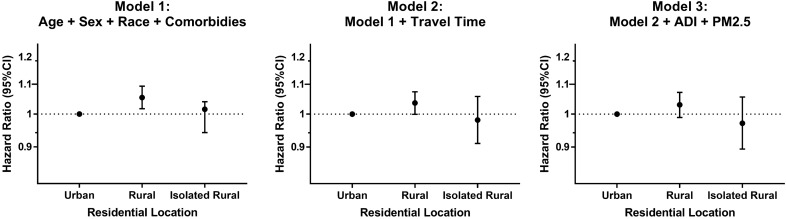
Association of residential location with mortality in patients who were alive after discharge following a second AECOPD-related hospitalization (n = 24,427). We created proportional hazards regression models to assess the association of residential location with mortality. All models included the following co-variates: obstructive sleep apnea, diabetes mellitus, congestive heart failure, coronary artery disease, cancer, and chronic kidney disease. AECOPD, acute exacerbation of COPD; HR, hazard ratio.

In the mortality analysis limited to COPD patients who were alive after discharge for their second AECOPD-related hospitalization (n = 24,427), mortality was 58.5% (8707 of 14,880) in urban patients, 59.1% (4871 of 8236) in rural patients, and 58.1% (762 of 1311) in isolated rural patients over a median follow-up time of 388 days (Interquartile Interval = 136–810 days). After adjusting for age, sex, race, and comorbidities, rural living was associated with increased mortality (hazard ratio (HR) = 1.05; 95% CI: 1.02–1.09, P = 0.004) but isolated rural living was not (HR = 1.02, 95% CI: 0.94–1.04, P = 0.70) (Fig. 3). After adding travel time to the model, both rural living (HR = 1.04; 95% CI: 0.99–1.07, P = 0.057) and isolated rural living (HR = 0.98, 95% CI: 0.91–1.06, P = 0.62) were not associated with mortality relative to urban living.

## Discussion

In a retrospective cohort study of COPD patients aged 65 or older enrolled in the VA health care system, we found that rural living was associated with lower risk for AECOPD-related hospitalizations, but isolated rural living was not associated with AECOPD-related hospitalizations after adjusting for demographics and comorbidities. Only when we adjusted for travel time to the closed VA medical center, isolated rural living was associated with more AECOPD-related hospitalizations. The inverse association of rural and isolated rural living with the “frequent exacerbator phenotype” went away after adjusting for travel time, neighborhood socioeconomic status, and air quality. A weak association between rural living and mortality disappeared when we accounted for travel time.

Approximately, one out of five Americans^27^ and more than a quarter of veterans reside in rural areas^24^. According to US Medicare data in 2015, there were 13.8 AECOPD-related hospitalizations per 1000 enrollees living in rural areas compared to 11.4 per 1000 enrollees living in urban areas^12^. Age-adjusted death rates due to COPD were 54.5 per 100,000 enrollees residing in rural areas relative to 32.0 per 100,000 enrollees residing in urban areas. Age-adjusted death rates in heart disease, stroke, cancer, and COPD are higher in rural areas relative to urban areas^28^. While the age-adjusted death rates in rural areas for heart disease, stroke, and cancer decreased or were similar between 2009 and 2014, the rates for COPD increased over time.

The burden of COPD is undoubtfully higher in rural areas than that in urban areas, but epidemiological studies cannot establish whether the disparity is related to a higher prevalence of COPD or worse outcomes of patients with COPD that live in rural areas. The average COPD prevalence in large urban areas is about 5% but the average prevalence in rural areas is 8% with some rural counties as high as 15.6%^12^. High smoking rates, in particular among rural veterans^29–31^, secondhand smoking exposure, and environmental and occupation exposures are important factors for higher COPD prevalence in rural areas and as a consequence of that, higher AECOPD-related hospitalization rates in rural areas relative to urban areas.

Analyzing data from the Subpopulations and Intermediate Outcome Measures in COPD Study (SPIROMICS), a multicenter US study, Burkes et al.^13^ examined the association of rural living and exacerbations. Among patients with COPD, rural living was an independent risk factor for moderate AECOPDs and this association persisted after accounting for lung function and demographics^13^. Nevertheless, an association of rural living and severe AECOPDs, defined as those who require emergency room visits and hospitalizations, was not observed. The SPIROMICS authors assumed that they did not observe any difference in severe exacerbations between urban and rural patients with COPD because of the small sample size and selection bias. In the SPIROMICS cohort, most participants were recruited in large urban academic centers. Thus, both rural and urban patients had access to those centers.

Our observation confirms Centers for Disease Control and Prevention (CDC) epidemiological data showing that isolated rural Medicare enrollees have more AECOPD-related hospitalizations than urban enrollees^12^. This association was observed only when travel time to the closest VA medical center was taken into account suggesting that other aspects than hospital care may be responsible for the excess of hospitalizations in isolated rural patients e.g. poor access to outpatient care or pulmonary rehabilitation^32–35^. Interestingly the counts of AECOPD-related and total hospitalizations (AECOPD and non-AECOPD) follow similar distribution between rurality groups (Supplement Table 1), which indicate that the gaps in care may not be COPD-specific. We did observe that rural patients have fewer AECOPD-related hospitalizations than urban patients. Based on the definition of rurality we used^24^, rural patients may refer to “suburban” residents who may have access to appropriate outpatient pulmonary care and live in an environment similar to that of urban areas. However, because they live further away from the hospitals may choose not to come the hospital until absolutely necessary as opposed to urban patients who may often seek care to the hospital due to easy access. On the contrary, isolated rural patients reside in very small communities with typical population < 2500 which lack resources for their care e.g. pulmonologists, pulmonary rehab. This may be the reason that isolated rural living was associated with more hospitalizations.

The “frequent exacerbator phenotype”, defined as those patients with ≥ 2 exacerbations per year has recently received attention as these patients consume disproportionate amounts of resources and are associated with worse outcomes^10,36^. Rural and isolated rural living were inversely associated with “frequent exacerbator phenotype” in the adjusted analysis that did not include travel time to the closet VA hospital, but the association went away when travel time, neighborhood socioeconomical status, and air quality. Galiatsatos and colleagues using SPIROMICS databased showed that disadvantaged neighborhoods (ADI) had higher rates of exacerbations and severe COPD exacerbations, defined as AECOPDs require emergency room visit or hospitalization^37^. Moreover, ambient PM_2.5_ is also associated with increased AECOPD-related hospitalizations^38^.

Death rates due to COPD are also higher among rural relative to urban residents according to epidemiological data^12^. The excess deaths in rural areas may result from the higher prevalence of COPD and comorbidities in residents living in those areas. In our analysis we found that rural living was associated with increased mortality relative to urban even after adjusting for comorbidities. We cannot rule out that the excess deaths in rural patients are because rural patients may choose to come to the hospital less frequently than urban patients. Surprisingly, isolated rural patients do not have increased long-term mortality relative to urban but this could be related to smaller sample size of isolated rural group. Nevertheless, after accounting for travel time, the association between rurality and mortality goes aways. Likely rural and urban patients receive similar care during AECOPD-related hospitalizations. Early studies showed that rural patients may have worse outcomes because they seek care at low volume hospitals^39,40^. However, with the widespread use of non-invasive ventilation, hospital volume may not play as an important role^41^. Our previous work showed that in-hospital mortality did not vary between rural and urban patients^16^.

Our study has some limitations. We conducted the study in single health care system with a predominantly male population. We included only COPD patients aged 65 or older. We have no data for smoking exposure or pulmonary function data to confirm COPD. However, our previous study showed 80–90% accuracy to identify AECOPD^16^ and we included only patients with at least two COPD encounters. Travel time to the closest VA hospital was calculated, but we did not have travel time to the closest community hospital. We have no data regarding the cause of the deaths. The above do not undermine the strengths of our study which include the large sample size, an adjustment for comorbidities, ADI, and air quality, and the fact that we captured admissions at both VA and non-VA hospitals.

In conclusion, rural living was associated with fewer but isolated rural was not associated with AECOPD-related hospitalizations after adjusting for demographics and comorbidities. Only after accounting for travel time to the closest VA medical center, isolated rural living was associated with more AECOPD-related hospitalizations suggesting that other aspects than hospital care may be responsible for the excess of hospitalizations in isolated rural patients e.g. poor access to outpatient care or pulmonary rehabilitation. Rural living was weakly associated with increased long-term mortality, but this association goes away after adjusting for travel time. Future research should focus on innovative ways to address gaps in care of in rural patients with COPD.

## Supplementary Information

Supplementary Tables.

## Data Availability

The data that support the findings of this study are available from United States Department of Veterans Affairs (VA) but restrictions apply to the availability of these data, which were used under license for the current study, and so are not publicly available. Data are however available from the corresponding authors, Spyridon Fortis, upon reasonable request and with permission of VA. Original VA funded datasets will be retained on VA servers behind VA firewalls. These data will be provided to interested parties following proper filing and verification of a Freedom of Information Act (FOIA) request and approval by the Privacy Officer. These data will be maintained as required by VA data retention policies. Maintenance of original datasets and/or programming code to create analytical datasets from large, centralized VA data sources will permit validation of results.
