# Supplementary material for: Association of rural living with COPD-related hospitalizations and deaths in US veterans
Source: Sci Rep. 2023 May 16;13:7887. doi: 10.1038/s41598-023-34865-7 (PMC10188568; doi:10.1038/s41598-023-34865-7)
Supplement: Supplementary file 1 — Supplementary Tables. [file 41598_2023_34865_MOESM1_ESM.docx]

**Association of Rural Living with COPD-related Hospitalizations and Deaths in US Veterans**

Spyridon Fortis MD, Yubo Gao PhD, Arianne K Baldomero MD MS, Mary Vaughan Sarrazin PhD, Peter J Kaboli MD MS.

**Supplement Table 1.** Count of non-VA AECOPD-related hospitalizations and count of total hospitalizations (AECOPD-related and non-AECOPD-related) in urban, rural, and isolated rural veterans with COPD.

| **Residential Location** | **Number of Veterans with COPD** | **Total (AECOPD-related and non-AECOPD-related) hospitalizations** | **AECOPD-related hospitalizations** | **Non-VA AECOPD-related hospitalizations** |
| --- | --- | --- | --- | --- |
| **Urban** | 89,610 (58.9%) | 461,323 (61.9%) | 88,106 (61.5%) | 30,941 (59.4%) |
| **Rural** | 53,204 (35.0%) | 243,741(32.7%) | 47,609 (33.2%) | 18,291(35.1%) |
| **Isolated Rural** | 9,251(6.1%) | 39,979 (5.4%) | 7,476 (5.2%) | 2,848(5.5%) |

**Supplement Table 2.** Factors associated with AECOPD-related hospitalizations in COPD patients (Univariate Analysis; n=152,065).

|  | **Univariate** | |
| --- | --- | --- |
|  | **RR (95%CI)** | **P value** |
| **Age, every 10 years** | 1.32 (1.31, 1.33) | <0.001 |
| **Female Sex** | 0.59 (0.56, 0.61) | <0.001 |
| **Race** |  |  |
| **White** | ref | ref |
| **Black** | 1.02 (1.01, 1.03) | <0.001 |
| **Other** | 0.87 (0.84, 0.90) | <0.001 |
| **Patient residential location** |  |  |
| **Urban** | ref | ref |
| **Rural** | 0.87 (0.86, 0.88) | <0.001 |
| **Isolated** | 0.97 (0.95, 0.98) | <0.001 |
| **Obstructive sleep Apnea** | 0.79 (0.78, 0.80) | <0.001 |
| **Diabetes Mellitus** | 0.96 (0.95, 0.96) | <0.001 |
| **Congestive Heart Failure** | 1.56 (1.55, 1.57) | <0.001 |
| **Coronary Artery Disease** | 1.16 (1.15, 1.17) | <0.001 |
| **Cancer** | 2.18 (2.17, 2.20) | <0.001 |
| **Chronic Kidney Disease** | 1.65 (1.63, 1.66) | <0.001 |
| **Travel Time to VA, every 30 min** | 0.97 (0.97, 0.97) | <0.001 |
| **Area Deprivation Index, every 10** | 0.98 (0.98, 0.98) | <0.001 |
| **Annual PM2.5, every 5ppm** | 0.94 (0.93, 0.94) | <0.001 |

We created generalized linear models with a **Poisson distribution** to assess factors associated with AECOPD-related hospitalizations.

AECOPD = acute exacerbation of COPD, RR = relative risk.

**Supplement Table 3.**  Factors associated with “frequent exacerbator phenotype” in COPD patients (Univariate Analysis; n=152,065).

|  | **Univariate** | |
| --- | --- | --- |
|  | **OR (95%CI)** | **P value** |
| **Age, every 10 years** | 1.16 (1.13, 1.18) | <0.001 |
| **Female Sex** | 0.85 (0.73, 0.98) | 0.023 |
| **Race** |  |  |
| **White** | Ref | ref |
| **Black** | 1.20 (1.14, 1.26) | <0.001 |
| **Other** | 1.08 (0.948,1.233) | 0.25 |
| **Patient residential location** |  |  |
| **Urban** | Ref | ref |
| **Rural** | 0.87 (0.84, 0.91) | <0.001 |
| **Isolated** | 0.84 (0.774, 0.90) | <0.001 |
| **Obstructive sleep Apnea** | 0.87 (0.83, 0.91) | <0.001 |
| **Diabetes Mellitus** | 0.96 (0.923, 0.99) | 0.011 |
| **Congestive Heart Failure** | 1.66 (1.61, 1.72) | <0.001 |
| **Coronary Artery Disease** | 1.16 (1.12, 1.20) | <0.001 |
| **Cancer** | 1.84 (1.769, 1.90) | <0.001 |
| **Chronic Kidney Disease** | 1.41 (1.36, 1.47) | <0.001 |
| **Travel Time to VA, every 30 min** | 0.97 (0.96, 0.97) | <0.001 |
| **Area Deprivation Index, every 10** | 0.99 (0.99, 1.00) | 0.080 |
| **Annual PM2.5, every 5ppm** | 1.11 (1.06, 1.15) | <0.001 |

We created generalized linear models with **a logit link** to assess factors associated with frequent exacerbator phenotype, defined as patients that had ≥ 2 hospitalizations/year.

AECOPD = acute exacerbation of COPD, OR = odds ratio
